# Oral cancer patients experience mechanical and chemical sensitivity at the site of the cancer

**DOI:** 10.1186/s12885-022-10282-3

**Published:** 2022-11-11

**Authors:** Caroline M. Sawicki, Malvin N. Janal, Samuel J. Nicholson, Angie K. Wu, Brian L. Schmidt, Donna G. Albertson

**Affiliations:** 1 Department of Pediatric Dentistry, New York University College of Dentistry, 421 First Avenue, Room 233W, New York, NY 10010 USA; 2 Department of Epidemiology & Health Promotion, New York University College of Dentistry, Room 301, 433 First Avenue, New York, NY 10010 USA; 3 Department of Oral and Maxillofacial Surgery, New York University College of Dentistry, 421 First Avenue, Room 233W, New York, NY 10010 USA; 4 Bluestone Center for Clinical Research, New York University College of Dentistry, 421 First Avenue, Room 233W, New York, NY 10010 USA; 5 NYU Oral Cancer Center, New York University College of Dentistry, 421 First Avenue, Room 233W, New York, NY 10010 USA

**Keywords:** Oral cancer, Oral cancer pain, Mechanical sensitivity, Capsaicin sensitivity

## Abstract

**Introduction:**

Oral cancer patients suffer severe chronic and mechanically-induced pain at the site of the cancer. Our clinical experience is that oral cancer patients report new sensitivity to spicy foods. We hypothesized that in cancer patients, mechanical and chemical sensitivity would be greater when measured at the cancer site compared to a contralateral matched normal site.

**Methods:**

We determined mechanical pain thresholds (MPT) on the right and left sides of the tongue of 11 healthy subjects, and at the cancer and contralateral matched normal site in 11 oral cancer patients in response to von Frey filaments in the range of 0.008 to 300 g (normally not reported as painful). We evaluated chemical sensitivity in 13 healthy subjects and seven cancer patients, who rated spiciness/pain on a visual analog scale in response to exposure to six paper strips impregnated with capsaicin (0–10 mM).

**Results:**

Mechanical detection thresholds (MDT) were recorded for healthy subjects, but not MPTs. By contrast, MPTs were measured at the site of the cancer in oral cancer patients (7/11 patients). No MPTs were measured at the cancer patients’ contralateral matched normal sites. Measured MPTs were correlated with patients’ responses to the University of California Oral Cancer Pain Questionnaire. Capsaicin sensitivity at the site of the cancer was evident in cancer patients by a leftward shift of the cancer site capsaicin dose-response curve compared to that of the patient’s contralateral matched normal site. We detected no difference in capsaicin sensitivity on the right and left sides of tongues of healthy subjects.

**Conclusions:**

Mechanical and chemical sensitivity testing was well tolerated by the majority of oral cancer patients. Sensitivity is greater at the site of the cancer than at a contralateral matched normal site.

**Supplementary Information:**

The online version contains supplementary material available at 10.1186/s12885-022-10282-3.

## Background

Oral cancer patients suffer severe chronic and mechanically-induced pain at the site of the cancer [1–3]. Pain degrades quality of life by interfering with eating, drinking and talking. Patients no longer experience pain after surgical removal of the cancer [4], suggesting that oral cancer pain is initiated and maintained in the cancer microenvironment.

Oral cancer pain is attributed to sensitization or activation of primary afferent neurons by mediators released from the cancer and microenvironment. Current research implicates two receptors on peripheral neurons – TRPV1 (transient receptor potential cation channel subfamily V member 1) and TRPA1 (transient receptor potential cation channel subfamily A member 1) [5]. In preclinical cancer models, expression of TRPV1 and TRPA1 is increased in the trigeminal or dorsal root ganglia [5], including in oral cancer models [6]. Antagonism or genetic knockout of the receptors variably relieves mechanical allodynia and thermal hyperalgesia.

Capsaicin, the spicy component of peppers, is an agonist of TRPV1. We have recognized that some oral cancer patients report the onset of heightened sensitivity and aversion to spicy foods. Sensitivity or aversion of rodents to drinking capsaicin solutions is a measure of sensitization of TRPV1 on peripheral neurons. Mice lacking *Trpv1* are non-responsive to capsaicin [7], whereas mice with increased expression of *Trpv1* due to genetic alterations or cancer show increased sensitivity to capsaicin [8, 9].

Few studies have quantified pain at the site of cancer in patients. In their recent review, Martland and colleagues [10] identified 18 reports in which quantitative sensory testing (QST) had been used to measure cancer associated pain. They reported that the majority of studies (*n* = 15) investigated chemotherapy-induced peripheral neuropathy (CIPN), which was characterized by increased thresholds to vibration, light touch (von Frey hairs) and pinprick stimuli. Only three studies measured cancer-associated pain – one studied peripheral neuropathy, one, cancer-induced bone pain and one, pain following breast cancer surgery. No studies were included in which testing was carried out to measure mechanical sensitivity at the site of the primary cancer prior to surgery. While capsaicin-evoked pain or detection thresholds have been studied in human subjects (reviewed in [11]), we have not identified any studies of capsaicin sensitivity in oral cancer patients, or in cancer patients in general.

We designed a pilot study to explore whether patients’ mechanical pain and chemical sensitivity can be measured at the site of the cancer. We hypothesized that mechanical and chemical sensitivity would be greater when measured at the cancer site compared to a contralateral matched normal site. Specifically, we proposed that von Frey fibers in the range of 0.008 to 300 g, which are not experienced as painful when applied to healthy, non-inflamed mucosa would be detected as painful by cancer patients at the site of the cancer, but not at a contralateral matched normal site. Similarly, we proposed that patient perception of pain when exposed to a series of capsaicin concentrations would be greater at the site of the cancer compared to a contralateral matched normal site.

## Methods

### Study populations

We evaluated mechanical sensitivity in three study cohorts; two independent oral cancer patient cohorts (cohort #1, *n* = 6 and cohort #2, *n* = 11) and a cohort of healthy subjects (n = 11). Participants were recruited and screened through the New York University (NYU) Oral Cancer Center. Eligible oral cancer patients were at least 18 years of age, not pregnant or lactating, had a diagnosis of oral cancer, and had not previously received chemotherapy or radiation treatment for cancer. Informed consent was obtained from each participant prior to study activities. Healthy subjects were eligible if they were at least 18 years of age, not pregnant or lactating, and in good general health as evidenced by medical history. All participants completed a demographic questionnaire that included age, sex, ethnicity, and race. Participants also completed a smoking and alcohol history questionnaire. In addition, oral cancer patients were questioned regarding development of sensitivity to spicy foods. Pathology reports were reviewed for information on primary tumor stage and nodal status. The study was carried out in accordance with the Code of Federal Regulations on the Protection of Human Subjects (45 CFR Part 46), the National Institutes of Health requirements for human subjects research and institutional research policies and procedures of the Institutional Review Board (IRB) at New York University. The Committee on Human Research at NYU Langone Medical Center approved this study (10–01261, 15 September 2020).

### Mechanical detection threshold (MDT) and mechanical pain threshold (MPT)

Testing used a set of von Frey monofilaments with 20 different diameters (Bioseb, Pinellas Park, FL, USA). The number of each filament (1.65 to 6.65) corresponds to a logarithmic function of the equivalent forces of 0.008 to 300.0 g. Subjects were asked to close their eyes. The filament was applied vertically to the test site and pressure was applied slowly until the filament bowed with a total contact time of 1–2 seconds. The von Frey filaments are not perceived as painful when applied to normal, non-sensitized (i.e., non-inflamed) skin. For cancer patients, tests were carried out first on a contralateral matched unaffected site, then on the cancer. If patients were taking pain medication, they were asked to refrain for 24 hours prior to sensitivity testing. For healthy subjects, von Frey filaments were first applied to the left lateral border of the tongue, then the procedure was repeated on the right side of the tongue. After each patient was examined filaments were disinfected with CaviCide (Metrex, Orange, CA, USA).

### Testing for MDT in healthy subjects

We measured MDT using two random ascending staircases. The force of the first von Frey filament in ascending order that was perceived by the participant was noted as the first suprathreshold value. Next, beginning with the force which had been noted, the von Frey filaments were applied in descending order until the participant could no longer perceive the force. This force was noted as the first infrathreshold value. This process was repeated within the area tested. The MDT was defined as the geometric mean of the suprathresholds and infrathresholds obtained with the two staircases. The staircases are shown in Supplementary Fig. A.

### Testing MPT in oral cancer patient cohorts #1 and #2

We assessed MPT in cohort #1 using the ascending method of limits technique. The MPT was defined as the geometric mean of the fiber preceding the fiber reported as painful and the painful fiber. The trials for each subject are shown in Supplementary Fig. B.

In cohort #2, we measured MPT using a random staircase method to avoid anticipation bias that could occur with the methods of limits technique used for cohort #1. Two ascending staircases were interleaved. Fibers from staircases 1 or 2 were presented in the order previously determined by the random number generator as for the healthy subjects. This order was used for testing both the cancer and the contralateral matched normal site. The fibers were presented in ascending order until the patient reported pain (the first suprathreshold), followed by presentation of fibers of lesser intensity. The first fiber not reported as pain was defined as the infrathreshold. In some patients, testing continued so that more than one suprathreshold and infrathreshold were obtained. The MPT was defined as the geometric mean of the suprathresholds and infrathresholds. The staircases are shown in Supplementary Fig. C.

### Capsaicin sensitivity testing

Capsaicin sensitivity testing was carried out approximately 10 min after mechanical sensitivity testing in cohort #2. Psychophysical studies of oral capsaicin detection report capsaicin detection thresholds on the order of 10^− 4^ – 10^− 3^ mM [11–14]. Taste strips (Sense Trading, Groningen, NL) were impregnated with capsaicin by dipping the strips for 10 seconds in serial dilutions (0.0, 10^− 3^, 10^− 2^, 10^− 1^, 1.0 and 10.0 mM) of a 100 mM stock solution of capsaicin (≥95%, Sigma-Aldrich Co., St Louis, MO, USA) in 100% ethanol. Strips were dried for 3 h at room temperature to allow evaporation of the ethanol.

The method of ascending limits was used to determine chemical response thresholds in patients and subjects. Starting with the lowest concentration, capsaicin impregnated taste strips were applied in ascending order to the test site. Each strip was applied for 10 seconds. Taste strips were presented at least 1 min apart to allow resolution of the response to the previous stimulus. Following application of each taste strip, patients and subjects were asked to rate the sensation on a visual analog scale (VAS) from 0 (no sensation) to 10 (greatest sensation imaginable). For patients, taste strips were first applied to the unaffected area (i.e.*,* contralateral, anatomically matched to the cancer site). After response thresholds were determined for the unaffected area, taste strips were applied to the center of the cancer site in patients. For healthy subjects, the right and left sides of the tongue were tested in the same manner as the patients. Capsaicin sensitivity was evaluated by comparing the area under the curve (AUC) of the VAS (response) with log capsaicin concentration, assigning 10^− 4^ mM to the 0 capsaicin concentration strip.

### Pain questionnaire

The University of California San Francisco Oral Cancer Pain Questionnaire (UCSFOCPQ) was developed and validated for measurement of oral cancer pain [4, 15]. The questionnaire asks patients to rate the intensity of their pain on a 0–100 point VAS in response to eight questions. The UCSFOCPQ was administered to cancer patients at a preoperative clinic visit before being prescribed analgesics for their oral cancer pain and before any treatment, as described previously [4, 15]. If patients were taking pain medication, they were asked to refrain for 24 hours prior to completion of the UCSFOCPQ and sensitivity testing.

### Statistical analysis

Hierarchical clustering was performed using ClustVis [16]. Data were analyzed using GraphPad Prism for Mac OSX (version 9.3.1, GraphPad, San Diego, CA, USA) and IBM SPSS v25.0 (IBM, Corp, Armonk, NY, USA). Comparison of means between groups used an independent samples t-test and comparisons within groups used a paired t-test. A *p*-value of <.05 was considered statistically significant.

## Results

### Patient cohorts

One cohort of healthy subjects and two cohorts of oral cancer patients were recruited from the NYU College of Dentistry and NYU Oral Cancer Center (Table 1). Healthy subjects were younger than cancer patients. The two cancer cohorts did not differ in age and other demographic characteristics. One patient in cohort #2 received neoadjuvant therapy; however, the patient completed sensitivity testing prior to therapy.

**Table 1:** Cohort characteristics

Characteristic	Oral cancer cohort #1 ***n*** = 6	Oral cancer cohort #2 ***n*** = 11	Healthy subjects ***n*** = 11	***p***-level
	Mean (SD)	Mean (SD)	Mean (SD)	
**Age (years)**	63.0 (13.2)	57.3 (18.4)	32.5 (7.2)	*p* = 0.0001^δ^
	**% (n)**	**% (n)**	**% (n)**	
**Gender**				
**Male**	33.3 (2)	63.6 (7)	54.5 (6)	*p* = 0.4565
**Female**	66.7 (4)	36.4 (4)	45.5 (5)	
**Self-reported ethnicity**				
**Hispanic/Latino**	0.0 (0)	27.3 (3)	0.0 (0)	*p* = 0.5342
**Not Hispanic/Latino**	100.0 (6)	72.7 (8)	100.0 (11)	
**Self-reported race**				
**White**	100.0 (6)	45.5 (5)	36.4 (4)	*p* = 0.5593
**African American**	0.0 (0)	27.3 (3)	0.0 (0)	
**Asian/Pacific Islander**	0.0 (0)	18.1 (2)	54.5 (6)	
**Other**	0.0 (0)	9.1 (1)	9.1 (1)	
Primary tumor stage*				
**cT1**	16.7 (1)	0.0 (0)	N/A	
**pT1**	50.0 (3)	45.5 (5)		
**pT2**	16.7 (1)	18.2 (2)		
**pT3**	16.7 (1)	9.1 (1)		
**pT4**	0 (0)	27.3 (3)		
**Nodal status**				
**N+**	33.3 (2)	27.3 (3)	N/A	
**N0**	16.7 (1)	36.4 (4)		
**Nx**	50.0 (3)	36.4 (4)		
**Cancer site**				
**Tongue**	100.0 (6)	72.7 (8)	N/A	
**Gingiva**	0.0 (0)	18.2 (2)		
**Floor of Mouth**	0.0 (0)	9.1 (1)		
**Tobacco use**				
**Previous/Never**	83.3 (5)	100.0 (11)	100.0 (11)	*p* = 0.6051
**Current**	16.7 (1)	0.0 (0)	0.0 (0)	
**Alcohol use**				
**Previous/Never**	16.7 (1)	27.3 (3)	54.5 (6)	p = 0.5342
**Current**	83.3 (5)	72.7 (8)	45.5 (5)	

^δ^indicates a significant difference between healthy subjects and both cancer cohorts

*pT1 stage assigned following neoadjuvant therapy in one case

### In healthy subjects, MDT does not differ between the left and right sides of the tongue

We first demonstrated in healthy subjects that the results of mechanical testing do not differ between sides of the tongue. We enrolled 11 healthy subjects and assessed detection of von Frey fibers on the left and right sides of the tongue (Supplementary Fig. A). No healthy subjects reported pain in response to the von Frey fibers ≤300 g. The mechanical detection threshold (MDT) for all subjects and sides of the tongue was (mean ± SD = 0.02 ± 0.01 g), similar to earlier reports [17, 18]. There were no differences within subjects with respect to the left and right MDTs, suggesting that should differences between cancer and non-cancer sites in cancer patients be detected, they could be attributed to disease state, rather than technical variability.

### Determination of the range of fibers detected as painful when applied at the site of the cancer

**Fig. 1:**
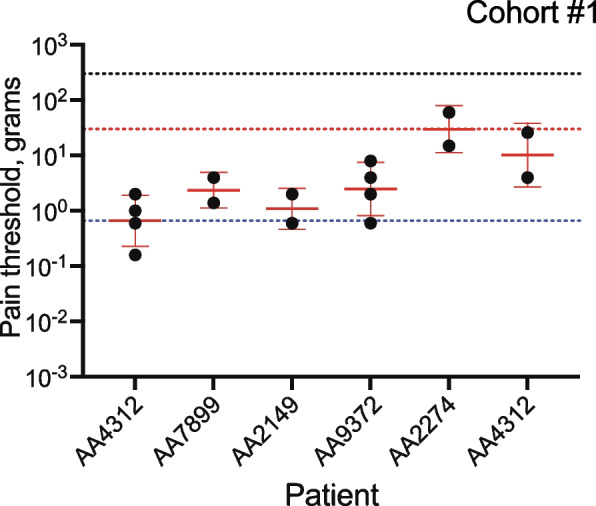
MPT in cohort #1. The MPT was defined as the geometric mean of pairs of fibers – the first fiber in a series reported as painful (suprathreshold) and the preceding fiber not reported as painful (infrathreshold). The geometric mean ± geometric SD is indicated in red. In two patients, testing continued such that two suprathresholds and two infrathresholds were obtained (Supplementary Fig. B). The black dotted line indicates the force corresponding to the maximum diameter fiber 6.65 and the red and blue dotted lines, the minimum and maximum measured MPTs

Patients in cohort #1 were tested for MPT at the site of the cancer by the ascending method of limits. In most cases, four filaments of increasing strength were tested (Supplementary Fig. B). The MPT ranged from 0.62 to 30 g (Fig. 1). These observations suggest that oral cancer patients experience pain in response to a range of von Frey filaments that normally do not evoke pain.

### Oral cancer patients report reduced MPTs at the site of the cancer

**Fig. 2:**
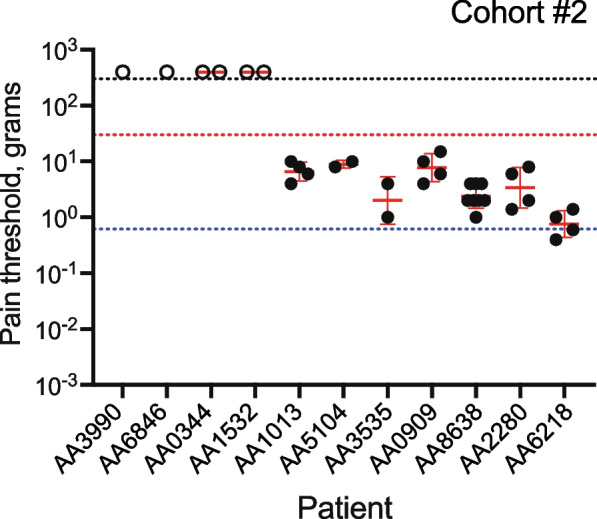
Oral cancer patients report pain at the site of the cancer in response to von Frey fibers. The MPT was defined as the geometric mean of the suprathresholds and infrathresholds. The geometric mean ± geometric SD is indicated in red. Open circles are arbitrary values indicating the four patients who did not report pain in response to any fiber including the largest diameter fiber, 6.65 (300 g). The black dotted line indicates the force corresponding to the maximum diameter fiber 6.65 and the red and blue dotted lines, the minimum and maximum measured MPTs in cohort #1

Based on our observations with cohort #1, we designed the study to ask if von Frey filaments typically used to assess MDT in healthy individuals will measure pain in oral cancer patients. We assessed MPT at the cancer and a contralateral normal site in 11 patients using a double random staircase method to avoid anticipation bias possible with the method of limits protocol applied with cohort #1 (Supplementary Fig. C). Seven patients reported pain in response to testing at the site of the cancer (Fig. 2). The MPTs were similar to the range established in cohort #1. By contrast, at the contralateral matched normal site, none of the 11 patients reported pain in response to testing, although patients reported detecting the fibers (Supplementary Fig. D).

### Oral cancer patients report greater sensitivity to capsaicin at the site of the cancer

**Fig. 3:**
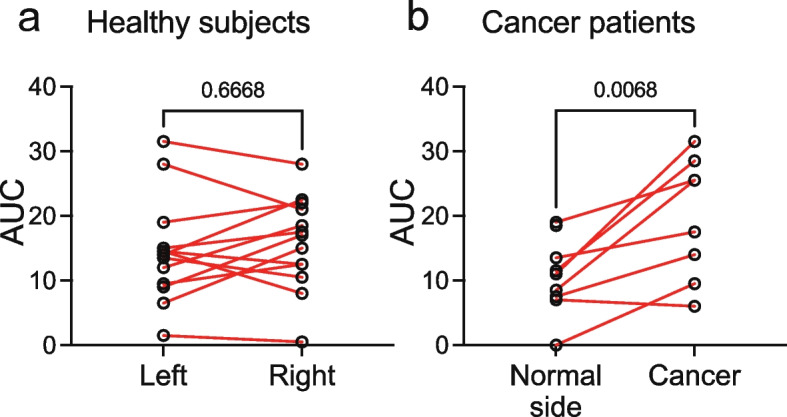
Cancer patients report increased sensitivity to capsaicin at the site of the cancer. **a** The area under the curve (AUC) of responses to increasing concentrations of capsaicin for the left and right sides of the tongue of healthy subjects did not differ. **b** The AUC of responses to increasing concentrations of capsaicin was greater at the site of the cancer compared to a matched contralateral normal site in cancer patients

Healthy subjects’ ratings increased in response to application of the paper strips impregnated with increasing concentrations of capsaicin (Supplementary Fig. E). Sensitivity to capsaicin increased linearly with the log_10_ capsaicin concentration (Supplementary Fig. F). The area under the curve (AUC) was determined for the dose-response plots of VAS with capsaicin concentration. There was no difference in AUC between the left and right sides of the tongue (Fig. 3a, *p* = 0.6668, two-tailed paired t-test).

Sensitivity to capsaicin was measured in nine of the 11 patients enrolled in cohort #2 (Supplementary Fig. G). Seven patients completed testing with all six strips on both the cancer and contralateral matched normal sites. One patient declined testing with the highest concentration of capsaicin on the cancer site due to sensitivity. The VAS score of the final strip tested (no. 5) was assigned. One patient refused testing on the cancer site due to pain and was not included in this analysis. The plots of VAS versus capsaicin concentration were shifted to the left for the cancer side compared to the contralateral matched normal site. The AUC for the cancer side dose-response plots was greater than the normal side (Fig. 3b, *p* = 0.0068, two-tailed paired t-test), indicating cancer associated sensitivity to capsaicin. We note, however, that in oral cancer patients the AUCs for the contralateral matched normal site dose-response plots were reduced relative to those of the healthy subjects (Fig. 3a and b). Factors contributing to this difference are unknown, but might include disease state, biological (e.g., intraoral anatomical differences in capsaicin sensitivity [19], and/or technical variation (e.g., test strip preparation batch effects).

### Patient-reported pain on the UCSFOCPQ correlates with the MPT measured at the oral cancer site

**Fig. 4:**
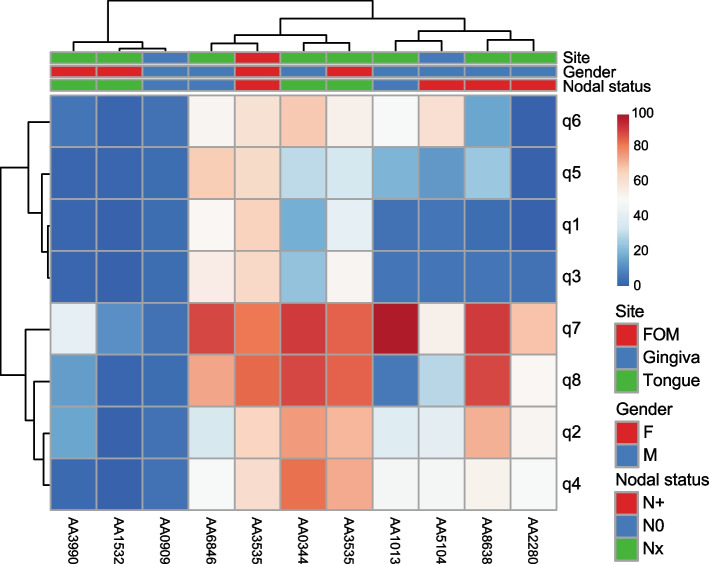
Responses of patients in cohort #2 on the UCSFOCPQ. Shown are patients in columns and responses to the UCSFOCPQ questions (q1-q8) in rows. Patients’ pain scores are distributed among low, medium and high responses to the eight questions, representing the range of responses observed in a larger cohort of patients [20]

All patients enrolled in the study completed the UCSFOCPQ prior to mechanical or capsaicin sensitivity testing. The UCSFOCPQ ask patients to rate on a visual analog scale how intense, sharp or aching is the pain when they are not eating, drinking or talking (questions 1, 3, 5), when they are eating, drinking or talking (questions 2, 4, 6), how sensitive is the area to touch (question 7) and how significantly does the pain restrict talking, eating or drinking (question 8). Patient responses clustered into two groups – 3 patients reported low pain in response to all questions and 8 patients reported varying levels of pain in response to the questions (Fig. 4). As reported previously, higher pain was reported in response to questions 7 and 8 and less pain in response to questions 1, 3 and 5, supporting the observation that oral cancer patients experience significant function-related pain prior to surgical resection of the cancer. The mean responses to the eight questions on the UCSFOCPQ from this small group were similar to the mean responses collected from a larger cohort of oral cancer patients (Mann-Whitney U Test, *p* = 0.16, 0.12, 0.23, 0.19, 0.28, 0.20, 0.39, 0.20, for questions 1–8, respectively) [20]. Spearman’s rank correlation was computed between the MPT and the eight UCSFOCPQ questions. There was a negative correlation between MPT and questions 2, 4 and 8 (r_s_ = −.79, *p* = 0.048; r_s_ = −.94, *p* = 0.005; r_s_ = −.79, p = 0.048, respectively) when considering only the seven patients for whom MPT was obtained. We note, however, that among the four patients for whom no MPT was measured, two women reported only low pain scores in response to all questions, while two men reported high scores. We found no association of the UCSFOCPQ with capsaicin sensitivity as measured by the AUC for the cancer side capsaicin dose-response plot or the difference in AUC of the dose-response plots between the cancer side and the contralateral matched normal site.

## Discussion

Mechanical and chemical sensitivity testing was well tolerated by the majority of oral cancer patients. Only two patients failed to complete capsaicin testing. All patients completed mechanical testing. We show that oral cancer patients report increased mechanical and chemical (capsaicin) sensitivity at the site of the cancer compared to a contralateral matched normal site in support of our hypothesis. Moreover, if MPT were measured at the cancer site, then the MPT correlated with the responses to UCSFOCPQ questions 2, 4 and 8, which measure pain when talking and eating and interference with functioning. By contrast, measures of capsaicin sensitivity did not correlate with the UCSFOCPQ. The questions on the UCSFOCPQ were designed to query functional pain and not chemical sensitivity. We note, however, that oral cancer patients are routinely questioned about sensitivity to spicy foods during clinical exam. Of the seven patients tested for capsaicin sensitivity, highest capsaicin sensitivity scores were recorded for the two patients who had reported sensitivity to spicy or acidic foods.

There are limitations to our study. The sample size is small; however, MPTs measured by two different methods in cohorts #1 and #2 were in the same range (no difference in means, *p* = 0.540, Welch’s two-tailed t test). Chemosensitivity testing was limited to evaluation of TRPV1 sensitivity by use of capsaicin, a TRPV1 agonist. Both TRPV1 and TRPA1 have been implicated in oral cancer and other pain syndromes [5]. Preclinical cancer pain models differ in impact of TRPV1 antagonism on mechanical allodynia and thermal hyperalgesia. TRPV1 and TRPA1 are co-expressed on trigeminal neurons and can interact to modulate receptor activity [21]. Studies are under way to quantify sensitivity of oral cancer patients to TRPA1 agonists. A more nuanced understanding of the contribution of chemical sensitivity, and the role of specific receptors and ion channels to oral cancer pain in patients might be obtained by assessing sensitivity to both TRPV1 and TRPA1. Additionally, due to the small sample size, we did not analyze the impact of tumor stage and comorbid conditions on cancer-induced sensitivity – studies we will undertake in the future, and when we have a larger oral cancer cohort. Our study was also limited to comparison of sensitivity within patients. Oral cancers display clinically visible alterations of the surface epithelium including erythematous and ulcerative changes. To better understand cancer specific mechanical and chemical sensitivity, future studies should measure sensitivity in patients with non-neoplastic ulcerative oral lesions, such as aphthous ulcers.

Oral cancer pain has a higher prevalence and severity greater than all other cancer pain [22]. Cancer pain has nociceptive and neuropathic components [23]. Inflammation appears to contribute less to oral cancer pain, as clinical and patient experience demonstrate that anti-inflammatory drugs do not alleviate the pain [24]. The density and type of neural innervation, along with the continuous need for oral function (e.g.*,* swallowing saliva, talking, eating, drinking) that stimulates the cancer area in the oral cavity, may contribute to the uniqueness, severity and high prevalence of oral cancer pain [25]. Sharp pain reported by patients at the site of the cancer [26] suggests cancer sensitizes or activates peripheral Aδ and C fibers that convey fast sharp pain and slow burning pain, respectively. Preclinical studies support the suggested sensitization of Aδ and C fibers. Oral cancer-induced sensitization of trigeminal nerves has been demonstrated using ex vivo single fiber recordings of lingual neurons in a murine orthotopic xenograft model [27]. Increased spontaneous firing of lingual nerves was recorded from the tongue cancer preparations. Reduced von Frey thresholds and mechanical hypersensitivity were observed for C- and A-slow-high-threshold mechanoreceptor (HTMR) fibers. No effects were observed on C-LTMR, A-slow-LTMR and A-fast lingual fibers. Cancer induced neuronal plasticity is further suggested by the observed decrease in mechanically insensitive afferent fibers in the ex vivo recording study [27], increased innervation of cancer by sprouting of neurites into the cancer microenvironment [28, 29] and changes in neuronal phenotype [28].

Oral cancer pain has been attributed to release from the cancer and/or cells of the cancer microenvironment of soluble mediators and extracellular vesicles carrying pain mediators with potential to sensitize TRPV1 and TRPA1 on sensory neurons [20, 30]. Pain mediators include lipids [31], ATP [32], nerve growth factor [33], proteases [34–38], cytokines [39, 40], genes involved in pain processing [41, 42] and micro RNAs [43, 44]. Patients with high scores on the UCSFOCPQ are at risk for metastasis [20]. Genes differentially expressed in metastatic cancers from patients reporting high pain are a subset of the meta-signature of the partial epithelial-to-mesenchymal transition (p-EMT) program [45, 46]. These p-EMT genes, expressed at the cancer/stroma interface, are well positioned to interact with neurites sprouting into the cancer microenvironment, and they include genes both known to be pain mediators and potential new mediators [20]. The p-EMT genes are a biomarker of oral cancer nodal metastasis [47].

Seven of the 11 patients reported pain in response to mechanical stimuli which did not evoke pain on their contralateral healthy sites. No MPT was measured for four patients. The absence of mechanical sensitivity in two cancer patients could be explained by their low UCSFOCPQ scores – the patients did not experience cancer pain. By contrast, the high pain scores on the UCSFOCPQ reported by the other two patients suggest that these patients are experiencing pain modalities not measured by von Frey testing. Preclinical studies of oral cancer pain find that cell lines differ in the composition of released mediators [48] and in the behavioral responses evoked by the mediators [31]. The compositions of released cancer pain mediators and their association with behavioral pain responses (e.g., mechanical, thermal, chemical sensitivity) have not been compared across cell lines. Differences in pain experienced by patients and differences in pain behavior displayed by animals in preclinical studies likely reflect differences between oral cancers – the variety of cancer subtypes identified, for example, by molecular profiling of the cancers and the immune microenvironment [49, 50]. The heterogeneity and plasticity of oral cancers highlights the need for sensory testing in patients in addition to studies in preclinical models.

## Conclusions

Mechanical pain and chemical sensitivity can be measured at the site of the oral cancer and are increased compared to a contralateral matched normal site. Mechanical and chemical (capsaicin) sensitivity testing together with preoperative assessment of pain with pain questionnaires might better inform clinicians of the pain experience by oral cancer patients at diagnosis.

## Supplementary Information

**Additional file 1.**

## Data Availability

The data supporting the conclusions of this article are included within the article and the additional files. The original case report forms are available from the corresponding author on reasonable request.

## Abbreviations

AUC: Area under the curve
MDT: Mechanical detection threshold
MPT: Mechanical pain threshold
TRPA1: Transient receptor potential cation channel subfamily A member 1
TRPV1: Transient receptor potential cation channel subfamily V member 1
UCSFOCPQ: University of California San Francisco Oral Cancer Pain Questionnaire
VAS: Visual analog scale
