# Supplementary material for: Oral cancer patients experience mechanical and chemical sensitivity at the site of the cancer
Source: BMC Cancer. 2022 Nov 11;22:1165. doi: 10.1186/s12885-022-10282-3 (PMC9650819; doi:10.1186/s12885-022-10282-3)

Supplementary Figures

Oral cancer patients experience mechanical and chemical sensitivity at the site of the cancer

Caroline M. Sawicki[1], Malvin N. Janal[2], Samuel J. Nicholson[3], Angie K. Wu[4], Brian L. Schmidt[3,4,5] and Donna G. Albertson[3,4,5]

Departments of [1]Pediatric Dentistry, [2]Epidemiology & Health Promotion and [3]Oral and Maxillofacial Surgery, [4]Bluestone Center for Clinical Research and [5]NYU Oral Cancer Center, New York University College of Dentistry

Supplementary Figure A. Staircases for healthy subjects. Filled circles = fiber detected.

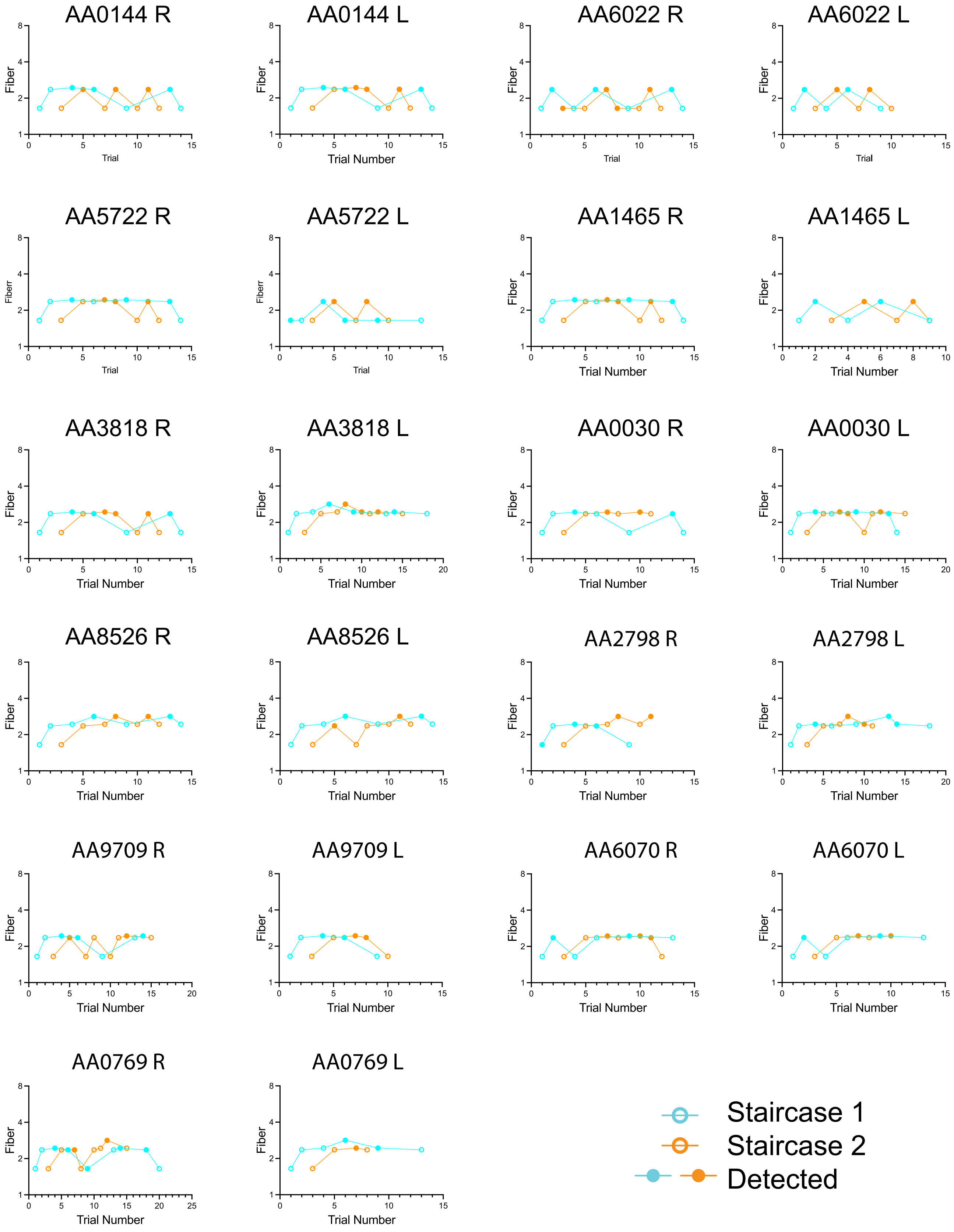

Supplementary Figure B. Testing regime for patients in cohort #1. Filled circles = fiber detected, filled circles with black boundary = fiber reported as painful. Black dotted line = maximum fiber, 6.65.

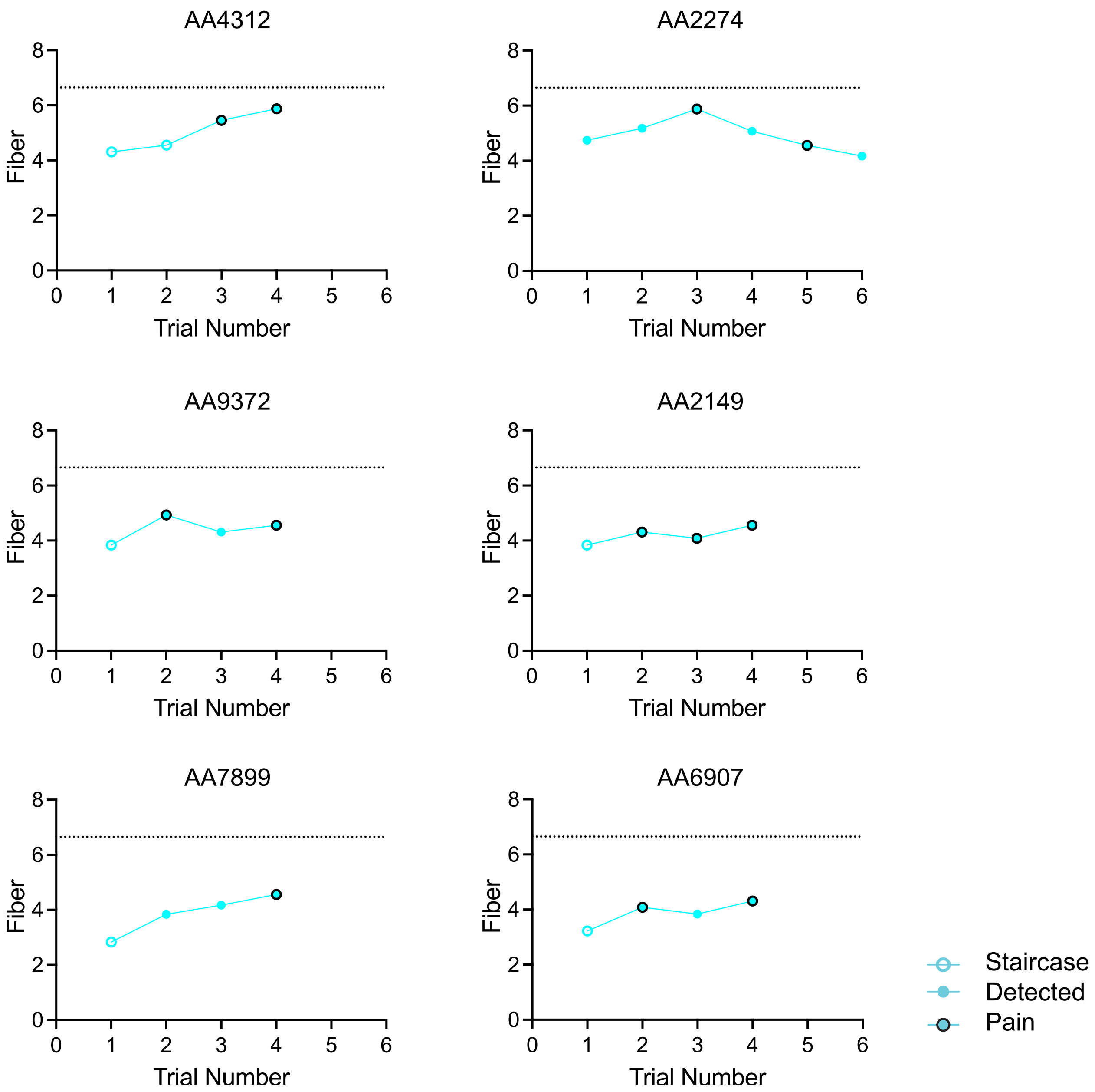

Supplementary Figure C. MPT measurement in cohort #2. Filled circles = fiber detected, filled circles with black boundary = fiber reported as painful. Black dotted line = maximum fiber, 6.65.

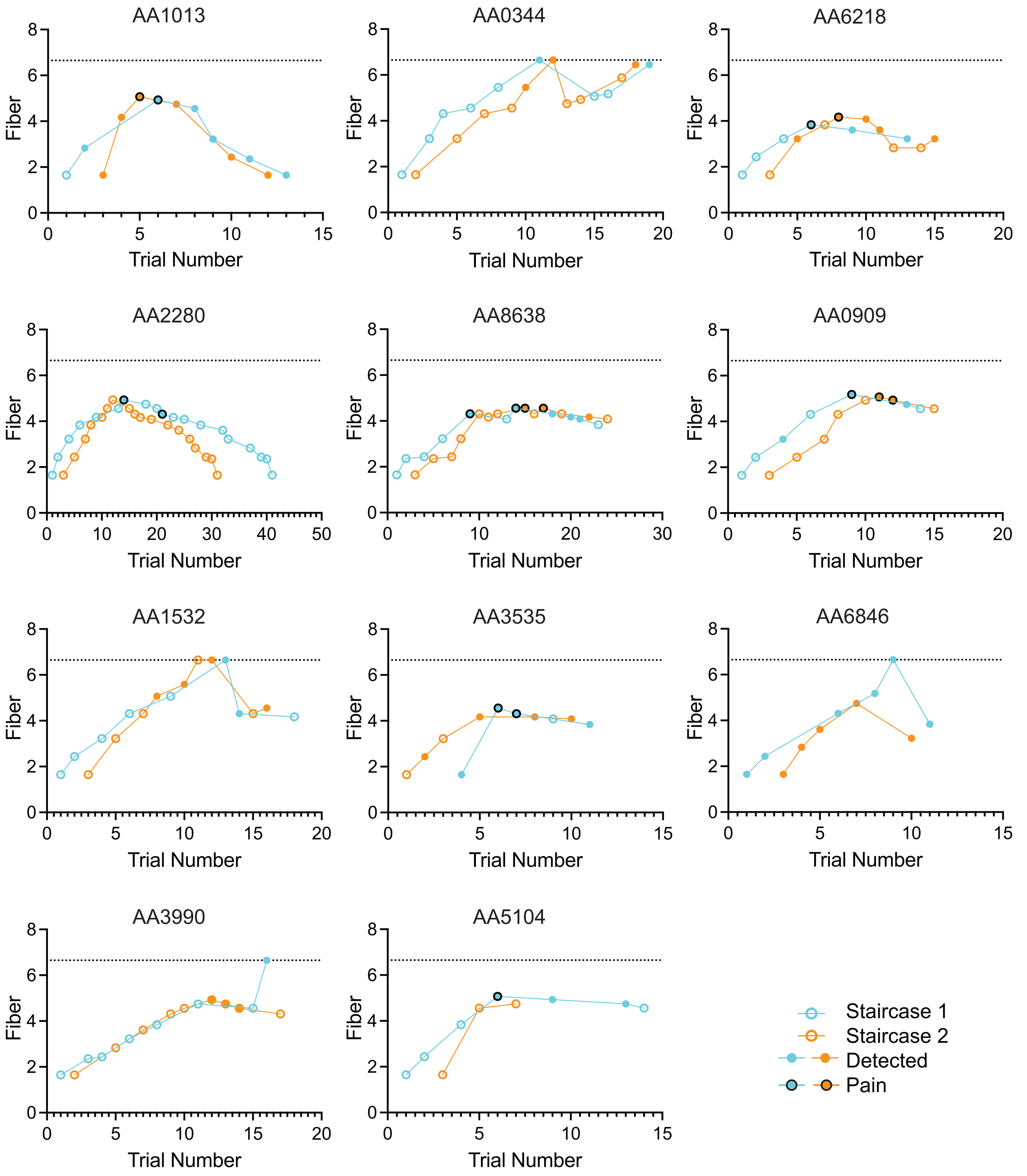

Supplementary Figure D. Staircases for testing the contralateral normal side of patients in cohort #2. Filled circles = fiber detected, filled circles with black boundary = fiber reported as painful. Black dotted line = maximum fiber, 6.65.

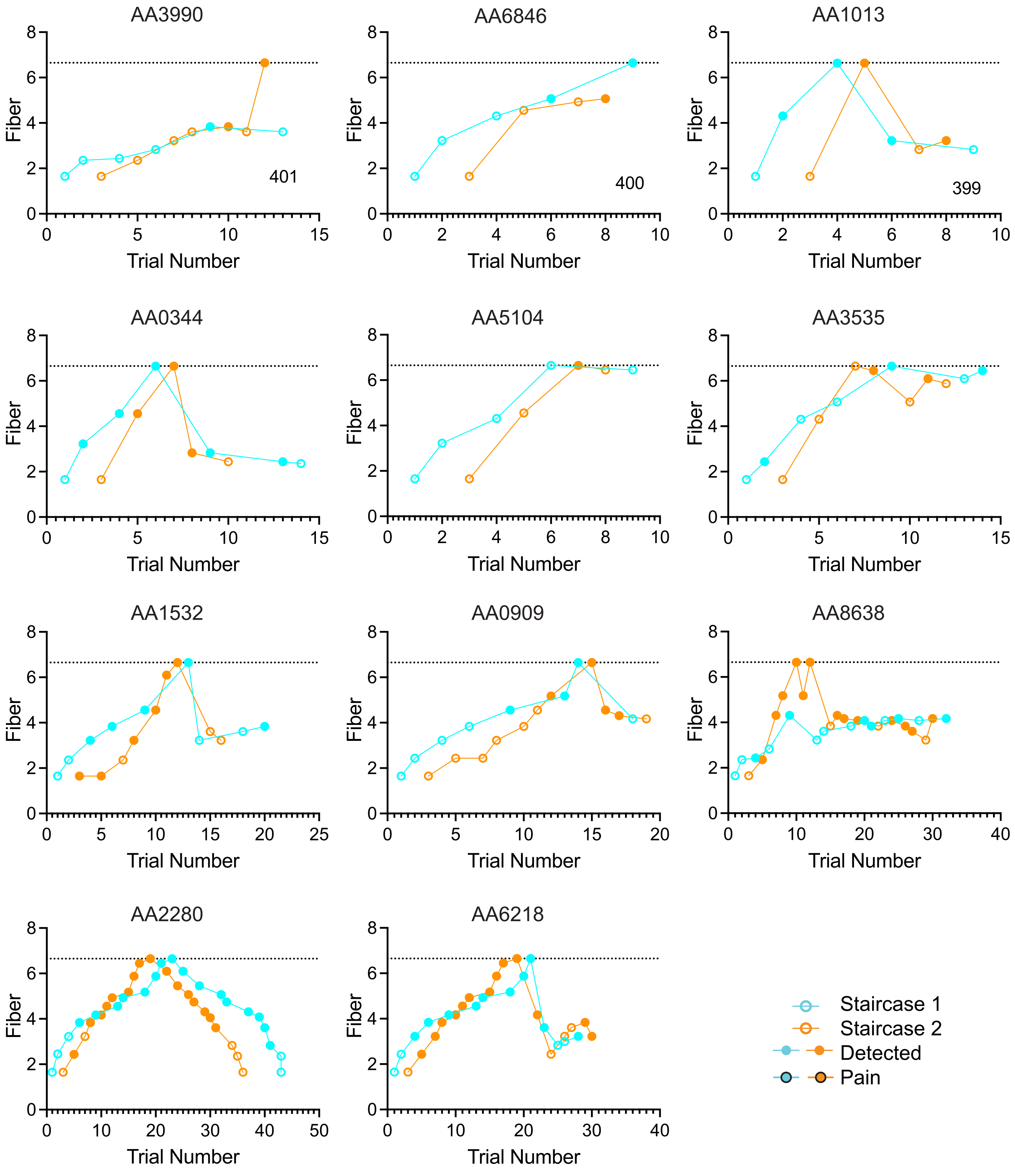

Supplementary Figure E. No difference in dose-response plots of VAS responses on the left and right sides of the tongue in healthy subjects.

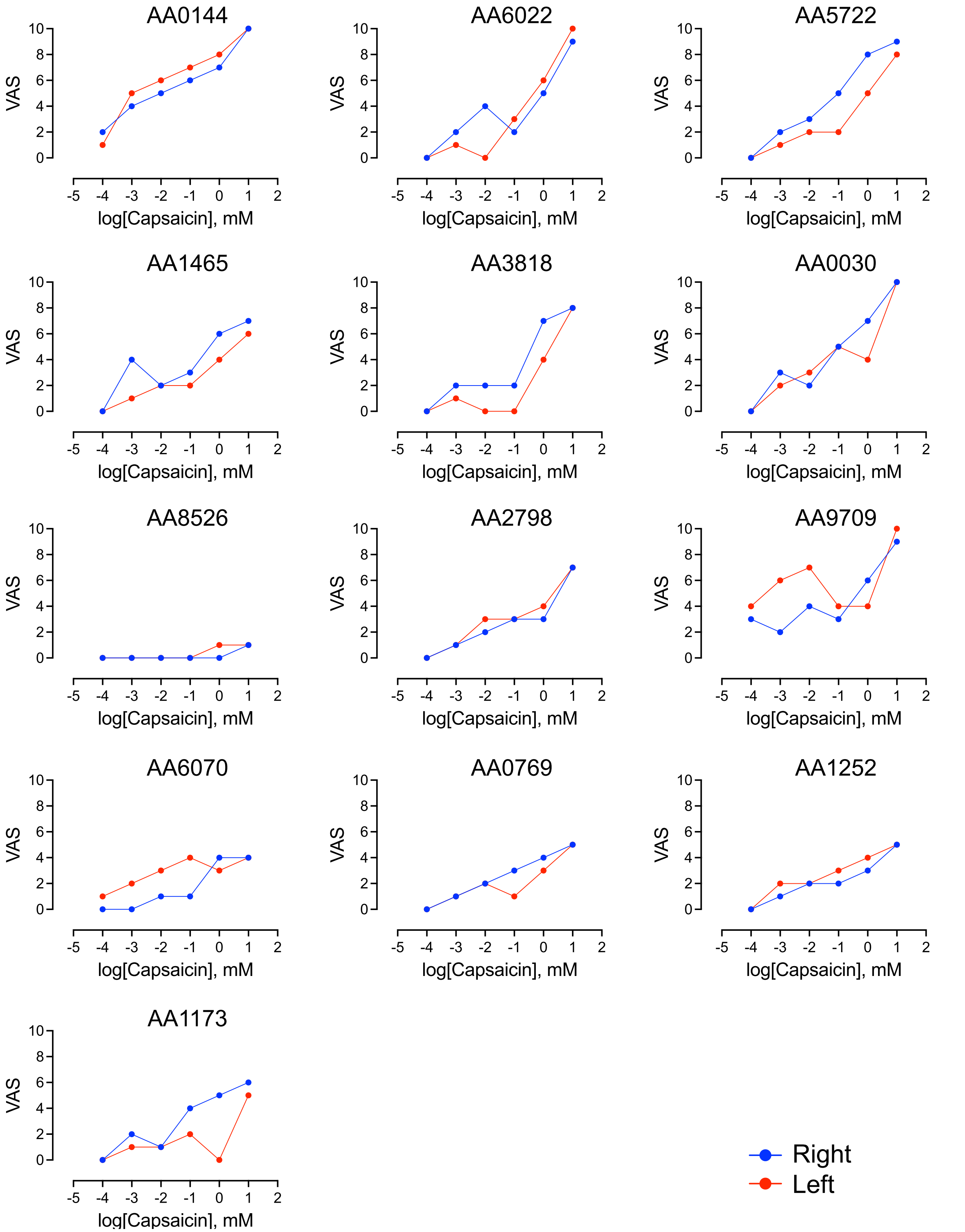

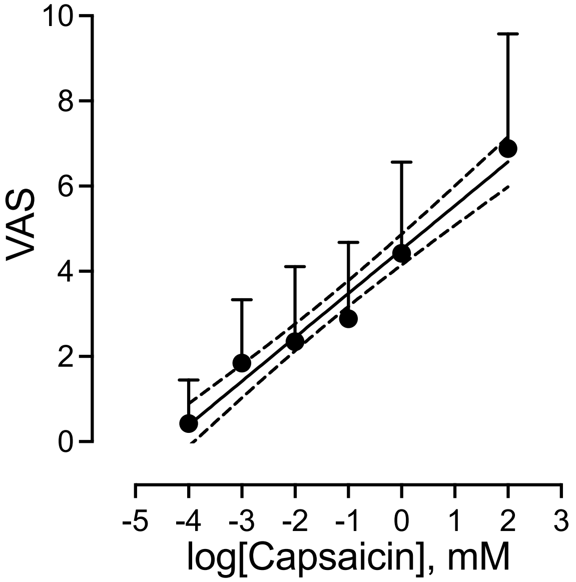

Supplementary Figure F. Visual analog scores increase with increased capsaicin concentration. Shown is the best fit line and 95% confidence intervals of the best fit line for the VAS from healthy subjects (mean + SD) for the left and right sides of the tongue (n=26 measurements). Simple linear regression was used to test if capsaicin concentration significantly predicted VAS. The fitted regression model was: y = 1.030x + 4.508. The overall regression was statistically significant (R^2 = 0.5383, F(1, 154) = 179.5, p<0.0001).

Supplementary Figure G. Dose-response plots of VAS responses of oral cancer patients are shifted to the left relative to dose-responses for testing on a contralateral matched normal site.

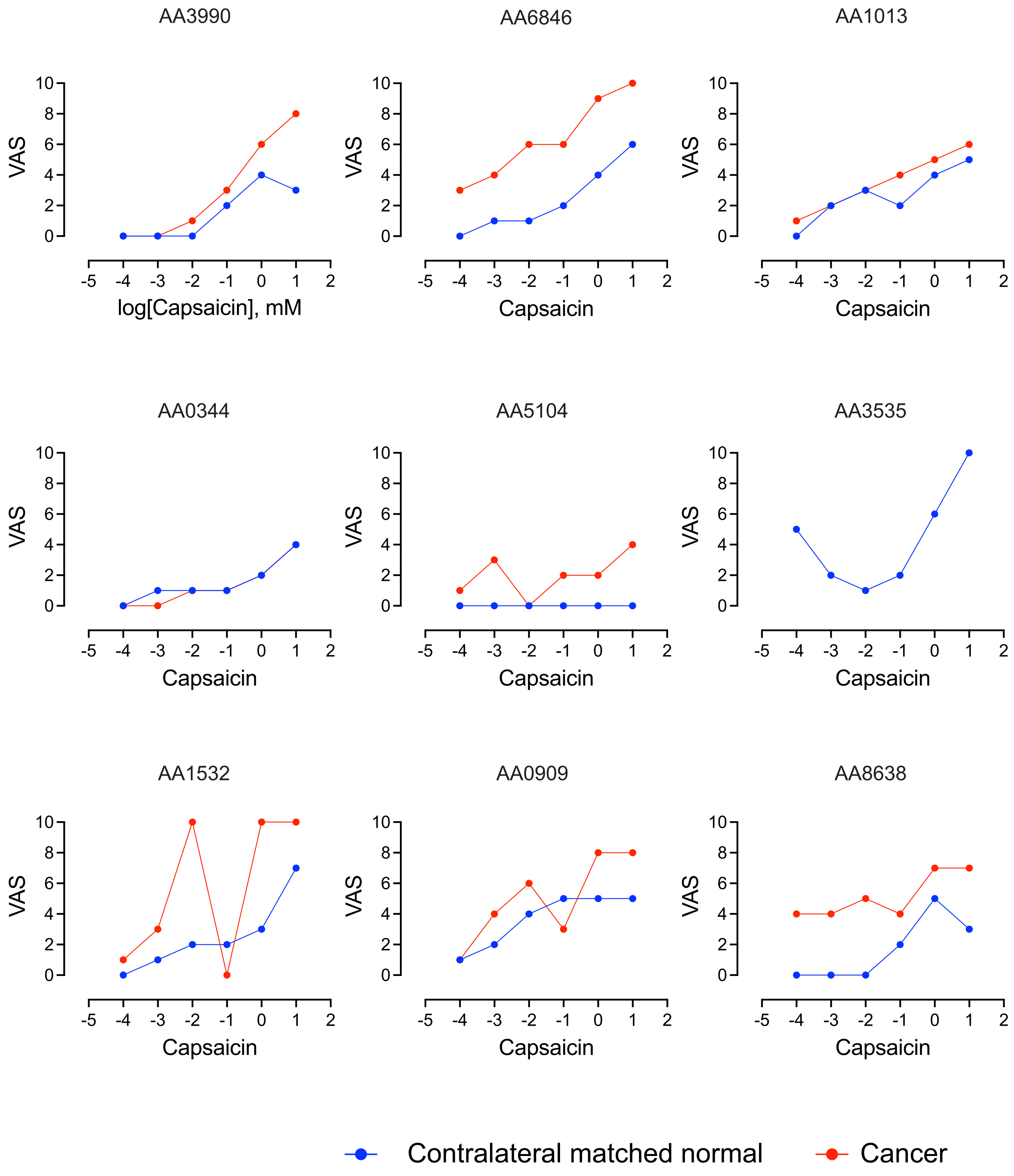

Supplement: Supplementary file 1 — Additional file 1. [file 12885_2022_10282_MOESM1_ESM.pdf]
